# Prevalence, virulence factor and antimicrobial resistance analysis of *Salmonella* Enteritidis from poultry and egg samples in Iran

**DOI:** 10.1186/s12917-021-02900-2

**Published:** 2021-05-24

**Authors:** Hassan Bahramianfard, Abdollah Derakhshandeh, Zahra Naziri, Reza Khaltabadi Farahani

**Affiliations:** 1 Department of Pathobiology, School of Veterinary Medicine, Shiraz University, Shiraz, Iran; 2 Department of Poultry Disease, National Reference Laboratories, Applied Studies and Diagnosis Center, Tehran, Iran

**Keywords:** Salmonellosis, *Salmonella* Enteritidis, Antibiotic resistance, Virulence genes, Poultry, Egg

## Abstract

**Background:**

*Salmonella enterica* serovar Enteritidis (*S*. Enteritidis) is one of the most common serovars, associated with human salmonellosis. The food-borne outbreak of this bacterium is mainly related to the consumption of contaminated poultry meat and poultry products, including eggs. Therefore, rapid and accurate detection, besides investigation of virulence characteristics and antimicrobial resistance profiles of *S*. Enteritidis in poultry and poultry egg samples is essential. A total of 3125 samples (2250 poultry and 875 poultry egg samples), sent to the administrative centers of veterinary microbiology laboratories in six provinces of Iran, were examined for *Salmonella* contamination, according to the ISO 6579 guideline. Next, duplex PCR was conducted on 250 presumptive *Salmonella* isolates to detect *invA* gene for identification of the genus *Salmonella* and *sdf* gene for identification of *S*. Enteritidis. Subsequently, the *S.* Enteritidis isolates were examined for detection of important virulence genes (*pagC*, *cdtB*, *msgA*, *spaN*, *tolC*, *lpfC*, and *spvC*) and determination of antibiotic resistance patterns against nalidixic acid, trimethoprim-sulfamethoxazole, cephalothin, ceftazidime, colistin sulfate, and kanamycin by the disk diffusion method.

**Results:**

Overall, 8.7 and 2.3% of poultry samples and 6.3 and 1.3% of eggs were contaminated with *Salmonella* species and *S.* Enteritidis, respectively. The *invA* and *msgA* genes (100%) and *cdtB* gene (6.3%) had the highest and the lowest prevalence rates in *S*. Enteritidis isolates. The *spvC* gene, which is mainly located on the *Salmonella* virulence plasmid, was detected in 50.8% of *S*. Enteritidis isolates. The *S*. Enteritidis isolates showed the highest and the lowest resistance to nalidixic acid (87.3%) and ceftazidime (11.1%), respectively. Unfortunately, 27.0% of *S*. Enteritidis isolates were multidrug-resistant (MDR).

**Conclusion:**

The rate of contamination with *Salmonella* in the poultry and egg samples, besides the presence of antimicrobial resistant and MDR *Salmonella* isolates harboring the virulence genes in these samples, could significantly affect food safety and subsequently, human health. Therefore, continuous monitoring of animal-source foods, enhancement of poultry farm control measures, and limiting the use of antibiotics for prophylactic purposes in food producing animals, are essential for reducing the zoonotic risk of this foodborne pathogen for consumers and also choosing effective antibiotics for the treatment of salmonellosis.

## Background

Salmonellosis is one of the most common zoonotic food-borne infections which is recognized as a major public health and economic problem worldwide [1]. *Salmonella enterica* serovar Enteritidis (*S*. Enteritidis) is the most common serovar, associated with human salmonellosis in many countries. Its food-borne outbreak is mainly due to the consumption of contaminated poultry and poultry products, including eggs [2]. The pathogenicity of salmonellae is associated with various virulence-encoding genes, located on the chromosome or the virulence-associated plasmid. For instance, fimbrial virulence genes, such as genes encoding long polar fimbriae (*lpf*), play a role in host recognition and mediate the adherence of bacteria to the intestinal epithelium and cellular invasion. Besides, the *invA* gene is involved in host recognition and invasion to the epithelial cells of intestinal mucosa. Some virulence genes, including *msgA, pagC,* and *tolC,* contribute to survival within the macrophage or intracellular survival. The *spaN* gene is associated with the invasive properties of *Salmonella* and facilitates entry into non-phagocytic cells and destruction of macrophages. Moreover, the *cdtB* gene is involved in host recognition and invasion and can induce apoptosis of infected cells by encoding the toxin [3, 4]. The *spvC* gene, which is mainly located on the virulence plasmid, plays a role in intracellular multiplication and survival of *Salmonella* within the host; it is also linked to systemic *Salmonella* infections [3, 5].

The clinical manifestations of *S*. Enteritidis range from self-limiting mild or moderate gastroenteritis to acute systemic infections that lead to mortality in high-risk patients [1].

Considering the extensive use or misuse of common antimicrobial agents in veterinary and human medicine for treating and preventing infections, besides their application for growth-promoting purposes, food-producing animals, especially poultry and their products, have become important reservoirs for drug-resistant bacteria [1, 6]. Moreover, drug-resistant bacteria, such as *S*. Enteritidis, can transfer from these animals to humans through the food chain, thereby limiting the antimicrobial treatment options for severe salmonellosis [7]. Accordingly, the healthcare costs have increased due to the increased rate and duration of hospitalization, treatment failure, and death among patients [6]. In this study, we aimed to investigate the prevalence, some virulence characteristics, and antimicrobial resistance profiles of *S*. Enteritidis in the poultry and poultry egg samples from six provinces of Iran.

## Results

### Phenotypic and genotypic identification of *S.* Enteritidis isolates

Overall, 250 (8.0%) *Salmonella* isolates were identified in 3125 poultry and poultry egg samples by phenotypic methods. Based on the results, 195/2250 (8.7%) poultry samples and 55/875 (6.3%) poultry egg samples contained *Salmonella* isolates. Among 250 *Salmonella* isolates, 63 (25.2%) were molecularly confirmed as *S.* Enteritidis. Thus 52/195 (26.7%) *Salmonella* isolates from poultry samples and 11/55 (20.0%) *Salmonella* isolates from poultry egg samples were identified as *S.* Enteritidis. The statistical analysis showed no significant difference in the frequency of *S.* Enteritidis isolates between the poultry samples and egg samples (*p* = 0.315). The total prevalence of *S.* Enteritidis isolates was 63/3125 (2.0%) in all samples, 52/2250 (2.3%) in poultry samples and 11/875 (1.3%) in poultry egg samples.

### Prevalence of virulence genes in *S.* Enteritidis isolates

Of eight studied virulence genes, *invA* and *msgA* genes, which were detected in all *S*. Enteritidis isolates, had the highest prevalence, while *cdtB* gene had the lowest prevalence in all *S*. Enteritidis isolates; the same result was obtained separately for *S*. Enteritidis isolates from poultry samples and poultry egg samples. The prevalence of virulence genes in *S*. Enteritidis isolates is presented in Table 1. The prevalence of *pagC* gene in *S*. Enteritidis isolates from poultry samples was significantly higher than that of *S*. Enteritidis isolates from poultry egg samples (*p* = 0.004). However, no significant difference was found in the prevalence of other tested virulence genes in *S*. Enteritidis isolates from poultry and egg samples (*p* > 0.05).

**Table 1:** The prevalence of virulence genes, virulence plasmid-associated gene, and antibiotic resistance among *S.* Enteritidis isolates^a^

	All ***S.*** Enteritidis isolates (***n*** = 63)	***S.*** Enteritidis isolates from poultry samples (***n*** = 52)	***S.*** Enteritidis isolates from egg samples (***n*** = 11)
**Virulence genes**			
*invA*	63 (100)	52 (100)	11 (100)
*pagC*	47 (74.6)	43 (82.7)	4 (36.4)
*cdtB*	4 (6.3)	4 (7.7)	0 (0.0)
*msgA*	63 (100)	52 (100)	11 (100)
*spaN*	49 (77.7)	41 (78.8)	8 (72.7)
*tolC*	49 (77.7)	41 (78.8)	8 (72.7)
*lpfC*	48 (76.1)	40 (76.9)	8 (72.7)
**Virulence plasmid-associated gene**			
*spvC*	32 (50.8)	25 (48.1)	7 (63.6)
**Antibiotic resistance**			
Nalidixic acid	55 (87.3)	46 (88.5)	9 (81.8)
Trimethoprim-sulfamethoxazole	13 (20.6)	12 (23.1)	1 (9.1)
vCephalothin	12 (19.0)	9 (17.3)	3 (27.3)
Ceftazidime	7 (11.1)	5 (9.6)	2 (18.2)
Colistin sulphate	15 (23.8)	13 (25.0)	2 (18.2)
Kanamycin	16 (25.4)	14 (26.9)	2 (18.2)

^a^Values are shown as number (%)

All *S*. Enteritidis isolates harbored at least two of the tested virulence genes (*invA* and *msgA*). The virulence score of all *S*. Enteritidis isolates (mean = 5.63, median = 6.0) and also *S*. Enteritidis isolates from poultry samples (mean = 5.73, median = 6.0) and poultry egg samples (mean = 5.18, median = 6.0) ranged from two to seven. The mean virulence score of *S*. Enteritidis isolates from poultry samples was significantly higher than that of *S*. Enteritidis isolates from poultry egg samples (*p* = 0.003).

Overall, ten different virulence profiles were observed in all *S*. Enteritidis isolates. Five profiles were only detected in *S*. Enteritidis isolates from poultry samples, one profile was only detected in *S*. Enteritidis isolates from poultry egg samples, and four profiles were common between *S*. Enteritidis isolates from poultry samples and egg samples. Nine and five different virulence profiles were detected in *S*. Enteritidis isolates from poultry samples and egg samples, respectively. The patterns of the presence of virulence genes in *S*. Enteritidis isolates are shown in Table 2.

**Table 2:** Patterns of virulence genes and antibiotic resistance in *S*. Enteritidis isolates^a^

	Number of virulence genes/ antibiotics	Number (%) in all ***S.*** Enteritidis isolates	Number (%) in ***S.*** Enteritidis isolates from poultry	Number (%) in ***S.*** Enteritidis isolates from eggs
**Virulence gene patterns**				
*invA, msgA, pagC, spaN, tolC, lpfC, cdtB*	7	1 (1.6)	1 (1.9)	0 (0.0)
*invA, msgA, pagC, spaN, tolC, lpfC,spvC*	7	20 (31.7)	18 (34.6)	2 (18.2)
*invA, msgA, pagC, spaN, tolC, cdtB*	6	1 (1.6)	1 (1.9)	0 (0.0)
*invA, msgA, pagC, spaN, tolC, lpfC*	6	16 (25.4)	14 (26.9)	2 (18.2)
*invA, msgA, spaN, tolC, lpfC,spvC*	6	11 (17.5)	7 (13.5)	4 (36.4)
*invA, msgA, pagC, cdtB*	4	1 (1.6)	1 (1.9)	0 (0.0)
*invA, msgA, pagC*	3	8 (12.7)	8 (0.0)	0 (0.0)
*invA, msgA, cdtB*	3	1 (1.6)	1 (1.9)	0 (0.0)
*invA, msgA, spvC*	3	1 (1.6)	0 (0.0)	1 (9.1)
*invA, msgA*	2	3 (4.8)	1 (1.9)	2 (18.2)
*-*	0	0 (0.0)	0 (0.0)	0 (0.0)
**Antibiotic resistance patterns**				
NAL, KAN, CST, SXT, CEF	5	1 (1.6)	1 (1.9)	0 (0.0)
NAL, KAN, CST, CEF, CAZ	5	6 (9.5)	5 (9.6)	1 (9.1)
NAL, KAN, CST, SXT	4	6 (9.5)	6 (11.5)	0 (0.0)
NAL, KAN, CST, CAZ	4	1 (1.6)	0 (0.0)	1 (9.1)
NAL, CST, SXT, CEF	4	1 (1.6)	1 (1.9)	0 (0.0)
NAL, KAN, SXT	3	1 (1.6)	1 (1.9)	0 (0.0)
KAN, SXT, CEF	3	1 (1.6)	1 (1.9)	0 (0.0)
NAL, SXT	2	1 (1.6)	1 (1.9)	0 (0.0)
SXT, CEF	2	2 (3.2)	1 (1.9)	1 (9.1)
NAL	1	38 (60.3)	31 (59.6)	7 (63.6)
CEF	1	1 (1.6)	0 (0.0)	1 (9.1)
-	0	4 (6.3)	4 (7.7)	0 (0.0)

^a^Values are shown as number (%)

### Distribution of virulence plasmid

Based on the detection of *spvC* gene, it can be concluded that almost half of all *S*. Enteritidis isolates harbored *Salmonella* virulence plasmid. The prevalence of virulence plasmid-associated gene (*spvC*) in *S*. Enteritidis isolates is presented in Table 1. The statistical analysis showed no significant difference in the prevalence of *spvC* gene and consequently, the distribution of virulence plasmids in *S*. Enteritidis isolates from poultry and egg samples (*p* > 0.05).

### Prevalence of antibiotic resistance in *S.* Enteritidis isolates

The highest prevalence of antibiotic resistance in all *S*. Enteritidis isolates and also separately in *S*. Enteritidis isolates from poultry and egg samples was reported against nalidixic acid. Resistance to ceftazidime in all *S*. Enteritidis isolates and also separately in *S*. Enteritidis isolates from poultry samples showed the lowest prevalence, whereas in *S*. Enteritidis isolates from poultry egg samples, the lowest prevalence of resistance was reported against trimethoprim-sulfamethoxazole. The prevalence of antibiotic resistance in *S*. Enteritidis isolates is presented in Table 1. The statistical analysis showed no significant difference in the prevalence of resistance to any of the tested antibiotics between *S*. Enteritidis isolates from poultry samples and poultry egg samples (*p* > 0.05).

The results showed that 93.7% of all 63 *S*. Enteritidis isolates were resistant to at least one of the tested antibiotics. Only four isolates from the poultry samples were not resistant to any of the six tested antibiotics, while all *S*. Enteritidis isolates from poultry egg samples were resistant to at least one of the tested antibiotics. None of the *S*. Enteritidis isolates were resistant to all of the tested antibiotics. Overall, the resistance score of *S*. Enteritidis isolates was in the range of 0–5 (mean = 1.90, median = 1.0) in the poultry samples and in the range of 1–5 (mean = 1.72, median = 1.0) in the poultry egg samples; however, no significant difference was found in the mean resistance score of these two groups (*p* = 0.092).

Overall, 11 different resistance profiles were observed in all *S*. Enteritidis isolates, six of which were only detected in *S*. Enteritidis isolates from poultry samples, two of which were only detected in *S*. Enteritidis isolates from poultry egg samples, and three of which were common between *S*. Enteritidis isolates from poultry and egg samples. Ten and five different resistance profiles were detected in *S*. Enteritidis isolates from poultry samples and egg samples, respectively. The patterns of antibiotic resistance in *S*. Enteritidis isolates are shown in Table 2. The most common resistance profile (60.3%) in all tested *S*. Enteritidis isolates was resistance to nalidixic acid alone. Unfortunately, 17 (27.0%) *S*. Enteritidis isolates were multidrug-resistant (MDR). No significant difference was found in the frequency of MDR between *S*. Enteritidis isolates from poultry samples (28.8%) and poultry egg samples (18.2%) (*p* = 0.712).

## Discussion

Contaminated poultry and eggs with non-typhoid *Salmonella,* especially *S*. Enteritidis, are the major sources of food-borne diseases in humans [6, 8]. Therefore, continuous monitoring of contamination in these animal-origin foods with salmonellae is necessary. Since the *invA* gene, which encodes the inner membrane protein, is only present and conserved in the genus *Salmonella* [9], we attempted to amplify this gold international marker to make a definite and rapid diagnosis of salmonellae in the samples [10]. Using this method, 8.7% of poultry samples and 6.3% of poultry egg samples, sent to the administrative centers of veterinary microbiology laboratories in six provinces of Iran, were found to be contaminated with *Salmonella*; this prevalence rate can be of major public health and economic importance for the country.

The rate of contamination of poultry samples with *Salmonella* was 3–66% in various epidemiological studies from different countries [9]. In this study, the rate of contamination of poultry egg samples with *Salmonella* was higher than the rates reported in some other studies, such as 0% in Cairo, Egypt [11], 0.3% in Dhaka, Bangladesh, 2.9% in Eastern Ethiopia, 3% in Belgium [12], 3.3% in North India [13], 3.8% in Tehran, Iran [14], and 5.40% in Guangdong, China [15]. However, the prevalence of *Salmonella* contamination of poultry eggs in the present study was lower than the rates reported in South India (7.7%), Nigeria (24.17%) [12], and Spain (34%) [16]. These differences in the rate of *Salmonella* contamination in poultry samples and egg samples can be related to differences in the hygienic control and management programs of different countries.

Considering the presence of discriminative *Salmonella* difference fragments (*sdf*) in chromosomes of *S. enterica* serovars [10], 26.7% of *Salmonella* isolates from poultry samples and 20.0% of *Salmonella* isolates from poultry egg samples were confirmed as *S.* Enteritidis. The results of other epidemiological studies conducted in 37 countries also revealed the importance of *S.* Enteritidis as the most prevalent serovar in contaminated poultry. For example, the prevalence of *S.* Enteritidis contamination in poultry samples was 19.2–49% in Africa and 5–93.7% in Asia and Europe [5].

Salmonellae have various virulence factors that contribute to their pathogenicity and increase the risk of serious infections in humans. The prevalence of *spvC* gene (50.8%) in the studied *S*. Enteritidis isolates was lower than that of chromosomally encoded virulence genes. This result was consistent with the findings of a study by Gritli et al., which reported a prevalence of 45.8% for *spvC* gene in *S*. Enteritidis isolates from chicken consumed in Tunisian military cantines [5]. However, this result contradicted the findings of a study that reported the higher prevalence of *spvC* gene (80%) in *S*. Enteritidis isolates [8] and the study indicating the lower prevalence of *spvC* gene (25.9%) in *S*. Enteritidis isolates [17].

In the present study, the *invA* and *msgA* genes were detected in all *S*. Enteritidis isolates and showed the highest prevalence among eight studied virulence genes. These results were consistent with the findings of other studies, which reported a prevalence of 100% for *invA* gene [4, 18–22] and *msgA* gene [19–21] in *Salmonella* isolates. The *cdtB* gene had the lowest prevalence as compared to other studied virulence genes, which is consistent with previous studies, reporting the low prevalence of this toxin-encoding gene [19–21]. Inequality of the virulence genes prevalence in *Salmonella* isolates of various studies can be due to genetic diversity and differences in pathogenicity of various *Salmonella* strains in different geographical regions.

The World Health Organization (WHO) surveillance programs indicate the *S*. Enteritidis as a principal foodborne pathogen in many countries [23]. In the past decades, the prevalence of resistant and MDR *S*. Enteritidis has increased globally, and poultry and poultry products are considered as a source of MDR *S*. Enteritidis in humans. Correspondingly, in our study, 28.8% of *S*. Enteritidis isolates from poultry samples and 18.2% of *S*. Enteritidis isolates from poultry egg samples were found to be MDR. This problem could limit the therapeutic options for infections, caused by antibiotic-resistant *S*. Enteritidis strains [6, 20, 24].

In the present study, the highest prevalence of antibiotic resistance (87.3%) in *S*. Enteritidis isolates was found against nalidixic acid. Also, the most common resistance profile (60.3%) in all tested *S*. Enteritidis isolates was resistance to nalidixic acid alone. The high prevalence of resistance to nalidixic acid was also reported in studies by Khaltabadi Farahani et al. (94.1%), En-Nassiri et al. (82%), and Ziyate et al. (61%) [8, 18, 22]. Conversely, in a study by Mezal et al., all *S*. Enteritidis isolates from poultry were sensitive to nalidixic acid. Besides, in a study by Han et al., resistance to nalidixic acid was only detected in 7.4% of *S*. Enteritidis isolates, and in a study by Gritli et al., resistance to nalidixic acid was seen in 16.66% of *Salmonella* isolates [5, 19, 20]. Since nalidixic acid is one of the recommended antibiotics for the treatment of *Salmonella* infections in humans, the high rates of nalidixic acid-resistant *S*. Enteritidis strains in poultry and poultry products are of great public health importance [25, 26]. The significance of this finding is related to the potential risk of transmission of these resistant strains to humans via consumption of poultry products, including poultry eggs [22].

On the other hand, fortunately in the present study, resistance to ceftazidime (11.1%), followed by cephalothin (19.0%), showed the lowest prevalence. Although these prevalence rates are not very low, the results are somewhat promising, as β-lactam antibiotics are suggested as the last option for the treatment of severe salmonellosis [25]. The low prevalence of resistance to β-lactam antibiotics (0–7%) has been also found in *S.* Enteritidis isolates in previous studies [5, 8, 27]. Conversely, Ghazaey and Mirmomeni reported that 90% of *S.* Enteritidis isolates from poultry samples were resistant to cephalothin [7]. Disparity in the prevalence and patterns of antibiotic resistance in *Salmonella* isolates of various studies may be due to difference in the amount and types of the prescribed antibiotics for prophylactic and therapeutic purposes and therefore different selection pressure in *Salmonella* strains of various geographical regions.

## Conclusion

The contamination of poultry and eggs samples with *Salmonella* in six provinces of Iran, besides the presence of antimicrobial-resistant and MDR *Salmonella* isolates harboring the virulence genes in these samples could highly impress on food safety and subsequently, human health. Therefore, continuous monitoring of animal-source foods, especially poultry meat and eggs, for the occurrence of contamination, antibiotic resistance patterns, and virulence characteristics of *Salmonella* is important to improve food safety, to reduce the zoonotic risk of this foodborne pathogen for consumers, and also to choose effective antibiotics for the treatment of salmonellosis. Based on the results, we recommend enhancing the poultry farm control measures, limiting the use of antibiotics (particularly those that are important in human medicine for prophylaxis purposes in food-producing animals), and informing the consumers of the importance of avoiding raw or undercooked poultry meat and eggs.

## Methods

### Ethics approval and consent to participate

This study was approved by the Animal Ethics Committee (AEC) of School of Veterinary Medicine, Shiraz University (code: MS 9234133). All animal experiments were performed in accordance with the guidelines and regulations of the AEC (September 20, 2013) and adhered to the Declaration of Helsinki.

### Sample collection and isolation of bacteria

During six months, a total of 3125 samples, including 2250 poultry samples and 875 poultry egg samples, were sent to the administrative centers of veterinary microbiology laboratories in six provinces of Iran (Tehran, Qazvin, Mazandaran, West-Azerbaijan, Khuzestan, and Sistan & Baluchestan). These samples were examined for the presence and identification of *Salmonella,* according to the international standard organization (ISO) 6579 guideline [27, 28]. Next, 250 presumptive *Salmonella* isolates were transferred to the Central Veterinary Laboratory of Iran Veterinary Organization and stored in a nutrient broth (Merck, Darmstadt, Germany), containing 20% glycerol at − 70 °C for further studies.

### Molecular confirmation and identification of presumptive *Salmonella* isolates

In the first step, DNA of presumptive *Salmonella* isolates was extracted using a High-Pure PCR Template Preparation Kit (Roche, Germany), according to the instructions. Next, a duplex polymerase chain reaction (PCR) assay was performed to detect *invA* gene for identification of the genus *Salmonella* and *sdf* gene for identification of *S.* Enteritidis. A positive control (*Salmonella* Enteritidis ATCC® 13076™) and a negative control were also included in the examination. The specific primer sequences and the PCR conditions are summarized in Table 3. Finally, the PCR products and DNA marker (CinnaGen Co., Iran) were resolved in 2% agarose gel (CinnaGen Co., Iran), containing ethidium bromide, and visualized under ultraviolet (UV) light of a transilluminator (UVitec, Cambridge, UK).

**Table 3:** Primer sequences, product sizes, and PCR conditions in this study

Target genes	Primer sequences (5′ to 3′)	Product size (bp)	PCR conditions	References
***Salmonella*** **genus specific gene**				
***invA***	**F:** AAACGTTGAAAAACTGAGGA **R:** TCGTCATTCCATTACCTACC	199	-Initial denaturation (95 °C for 10 min) − 30 cycles of: - Denaturation (94 °C for 60 s) - Annealing (62 °C for 90 s) - Extension (72 °C for 90 s) - Final extension (72 °C for 10 min)	[29]
***S.*** **Enteritidis serovar specific gene**				
***Sdf***	**F:** AAATGTGTTTTATCTGATGCAAGAGG **R:** GTTCGTTCTTCTGGTACTTACGATGAC	299		[30]
**Virulence genes**				
***pagC***	**F:** CGCCTTTTCCGTGGGGTATGC **R:** GAAGCCGTTTATTTTTGTAGAGGAGATGTT	454	-Initial denaturation (95 °C for 10 min) − 35 cycles of: - Denaturation (94 °C for 40 s) - Annealing (62 °C for 30 s) - Extension (72 °C for 40 s) - Final extension (72 °C for 10 min)	[21]
***cdtB***	**F:** ACAACTGTCGCATCTCGCCCCGTCATT **R:** CAATTTGCGTGGGTTCTGTAGGTGCGAGT	268		
***msgA***	**F:** GCCAGGCGCACGCGAAATCATCC **R:** GCGACCAGCCACATATCAGCCTCTTCAAAC	189		
***spaN***	**F:** AAAAGCCGTGGAATCCGTTAGTGAAGT **R:** CAGCGCTGGGGATTACCGTTTTG	504		
***tolC***	**F:** TACCCAGGCGCAAAAAGAGGCTATC **R:** CCGCGTTATCCAGGTTGTTGC	161		
***lpfC***	**F:** GCCCCGCCTGAAGCCTGTGTTGC **R:** AGGTCGCCGCTGTTTGAGGTTGGATA	641		
**Virulence plasmid-associated gene**				
***spvC***	**F:** ACTCCTTGCACAACCAAATGCGGA **R:** TCTCTTCTGCATTTCGCCACCATCA	571	- Initial denaturation (94 °C for 5 min) −35 cycles of: - Denaturation (94 °C for 30 s) - Annealing (57 °C for 40 s) - Extension (72 °C for 30 s) - Final extension (72 °C for 10 min)	[31]

### Detection of virulence genes

A total of 63 molecularly confirmed *S.* Enteritidis isolates, which were recovered from poultry and poultry egg samples, were examined to detect important virulence genes. For this purpose, two sets of multiplex PCR were designed for amplification of *pagC*, *cdtB*, and *msgA* genes and amplification of *spaN*, *tolC*, and *lpfC* genes. The specific primer sequences of virulence genes and the PCR conditions are summarized in Table 3. After termination of the amplification process, the PCR products, along with the DNA marker, were electrophoresed and visualized under the UV light of a transilluminator.

### Investigation of the distribution of virulence plasmid

To investigate the distribution of virulence plasmid in *S.* Enteritidis isolates based on the presence of *spv* (*Salmonella* plasmid virulence) locus, a PCR assay was designed for amplification of the virulence plasmid-associated gene, called *spvC*. The specific primer sequences of *spvC* gene and the PCR conditions are summarized in Table 3. The PCR products, along with the DNA marker, were electrophoresed and visualized under the UV light of a transilluminator.

### Determination of antibiotic resistance profiles

The antibiotic resistance patterns of 63 molecularly confirmed *S.* Enteritidis isolates against nalidixic acid, trimethoprim-sulfamethoxazole, cephalothin, ceftazidime, colistin sulfate, and kanamycin were determined by the disk diffusion method and interpreted according to the Clinical and Laboratory Standards Institute (CLSI) guidelines [32]. *Escherichia coli* ATCC® 25,922 was also included as a quality control [32].

### Statistical analysis

Statistical analysis and comparison of data were performed, using t-test, Chi-square test, and Fisher’s exact test in SPSS version 16.0 (SPSS Inc., Chicago, IL, USA). A *p* ≤ 0.05 was considered to be statistically significant.

## Data Availability

The data sets used and/or analyzed during the current study are available from the corresponding author on reasonable request.

## Abbreviations

*S*. Enteritidis: *Salmonella enterica* serovar Enteritidis
ISO: International Organization for Standardization
UV: Ultraviolet
NAL: Nalidixic acid
SXT: Trimethoprim–sulfamethoxazole
CEF: Cephalothin
CAZ: Ceftazidime
CST: Colistin sulfate
KAN: Kanamycin
PCR: Polymerase chain reaction
